# Accuracy of Prenatal Diagnosis in Elective Termination of Pregnancy: 385 Cases from 2000 to 2007

**DOI:** 10.5402/2011/458120

**Published:** 2010-11-08

**Authors:** Fabiana Ramos, Sofia Maia, Miguel Branco, Joana Raposo, Joaquim Sá, Sérgio Sousa, Margarida Venâncio, Raquel Pina, Eulália Galhano, Lina Ramos, Jorge Saraiva

**Affiliations:** ^1^Serviço de Genética Médica, Centro Hospitalar de Coimbra, Avenida Bissaya Barreto, 3000-076 Coimbra, Portugal; ^2^Centro de Diagnóstico Pré-natal, Centro Hospitalar de Coimbra, 3000-061 Coimbra, Portugal; ^3^Serviço de Anatomia Patológica, Centro Hospitalar de Coimbra, 3041-853 Coimbra, Portugal

## Abstract

*Objective*. To evaluate the quality of prenatal results in all cases of termination of pregnancy (TOP) due to fetal abnormalities in a tertiary prenatal diagnosis center. *Material and Methods*. Retrospective analysis of the 385 TOP performed on our department due to fetal abnormalities between January 1, 2000, and December 31, 2007. We compared all data for agreement between the ultrasound, genetic, and postmortem findings, regarding the abnormalities identified in the etiological diagnosis and its prognosis. *Results*. Chromosome abnormalities were the most common indication for TOP (39%), followed by abnormalities of CNS (20%), monogenic disorders (11%), sequences (9.6%), polimalformative syndromes (5.2%), and isolated congenital heart diseases (4%). Total agreement was 21%. Further abnormalities were identified in 79%. The data collected after TOP changed the etiologic diagnosis in 21% but the prognosis was changed in only one fetus. *Discussion*. This study corroborates the necessity of a multidisciplinary team in prenatal diagnosis centers. Their work remarkably improves the genetic counseling and represents an important aspect in quality control of the information given to a couple previously to a TOP.

## 1. Introduction

Fetal abnormalities are the major cause of perinatal death and contribute to the increase of the morbidity after birth [1, 2]. In this context prenatal ultrasound has become the main method for detecting fetal anomalies [3, 4].

Accurate prenatal diagnosis of fetal anomalies is essential to the parental decision and the assessment of the recurrence risk. The genetic evaluation and fetal pathology examination after the TOP allow to improve the ability to achieve a diagnosis, to refine the prognosis, and to enhance the genetic counseling, as well as to audit the quality and accuracy of prenatal ultrasound [5–8].

The main goal of this study was to review the prenatal diagnosis experience in cases of TOP for fetal abnormalities in our department over a period of eight years. We have collected and compared the prenatal clinical findings with the genetic evaluation and the postmortem results. This was done in order to assess the contribution of genetic evaluation and autopsy in improving the diagnostic skills and the couple's counseling.

## 2. Material and Methods

We audited 385 cases of TOP performed because of fetal abnormalities between January 1, 2000, and December 31, 2007, at the Prenatal Diagnosis Center of Maternidade Bissaya Barreto, Centro Hospitalar de Coimbra. All cases had data regarding the results of prenatal ultrasound and laboratory evaluation, observation of the fetus after TOP by clinical geneticist, who could request further exams (such as fetal X-rays cytogenetic, molecular, or enzymatic tests of fetal samples) and fetal pathology. 

All cases with fetal malformations were evaluated for the same team of sonographers, trained in fetal medicine. The ultrasound equipment used was a Toshiba Xario that has transabdominal and transvaginal transducers with frequencies ranging from 3.5 to 5 MHz.

Cytogenetic (karyotype and FISH analysis for specific probes) and molecular tests were performed when indicated after amniocentesis or chorionic villus sampling biopsy. In many cases with cytogenetic abnormalities, invasive prenatal diagnosis was carried out due to advanced maternal age (age greater or equal to 35 years at the estimated due date). 

The postmortem studies were performed after informed consent and were done according to protocols described by Keeling and adapted by Brandão and Laurini [9, 10]. The ultrasound and genetic findings were available to the pathologists before the postmortem evaluation. 

All TOPs performed at our department were consecutively recorded. We reviewed the TOP due to fetal abnormalities and gathered all the data from the sonographic, genetic (fetal observation and laboratorial tests), and postmortem records. The results were compared for the presence or absence of agreement at the TOP and the final genetic counseling. Total agreement was considered when no relevant findings (regarding abnormalities that threaten life, require surgery or medical treatment, impair significantly organic function or appearance or were relevant to the etiological diagnosis) were added to ultrasound results after genetic evaluation or autopsy. Disagreement was stated when relevant findings were identified at the genetic and/or fetal pathology evaluation.

Whenever disagreement was identified, we also evaluated whether it led to a change in the diagnosis and/or prognosis.

All the information was used to improve the genetic counseling of these couples.

## 3. Results

A total of 385 cases were included in this study. Chromosome abnormalities were the most common indication for TOP (149/385; 39%). Numerical chromosome abnormalities were diagnosed in 122 fetuses in which trisomy 21 was the leading cause (67 cases) followed by trisomy 18 (21 cases), trisomy 13 (16 cases), X monosomy with hydrops (12 cases), and mosaics for other numerical abnormalities (6 cases). Euploidies occurred in 5 cases (4 triploidies and 1 tetraploidy). Structural unbalanced abnormalities were presented in 22 cases. In 52% (77/149) of the chromosome abnormalities, the invasive prenatal diagnosis was performed due to advanced maternal age.

Central nervous system malformations were diagnosed in 77 cases (20%). Neural tube defects (anencephaly, myelomeningocele and encephalocele) were found in 59 cases (15%). A central nervous tumor was diagnosed in two fetuses. One was an embryonic tumor and the other a giant dermoid cyst.

Monogenic disorders were responsible for 11% of TOP (44/385 cases), which could be divided in two groups: those with previous familial history and those in which the fetus was the index case. On the first group, the molecular diagnosis was offered to 13 cases (3.4%). The analysis of an autosomal recessive disease was the most common request. In one couple with a previous child with Walker-Warburg syndrome, the diagnosis was exclusively based on ultrasound findings, because no mutation was found on the index case. Thirty-one cases (8%) belonged to the second group. On these disorders and after the fetal observation and postmortem findings, the genetic assessment allowed an accurate diagnosis in 84% (26/31 cases) of the cases. Their work was crucial in 16 of these cases, the pathological study by itself in five cases of them and a collaboration of both areas in other five cases.

Sequences (defined as a pattern of developmental anomalies consequent upon a primary defect, often with heterogeneous etiology) were presented in 37 cases (9.6%). The most common sequence was oligohydramnios sequence (Potter syndrome) in 17 cases. Amnion rupture sequence (amniotic bands) was diagnosed in six cases and early urethral obstruction sequence in also six cases; laterality sequences with left isomerism (polysplenia syndrome) and right isomerism (Ivemark syndrome) were identified in two cases. Other sequences were identified in six cases.

Twenty cases (5.2%) had a recognized nonmonogenic nonchromosomal syndrome. In all of these cases, no specific etiologic diagnosis could be found; however the observation of the fetus by the geneticist and the autopsy added essential information for the genetic counseling. 

Sixteen cases (4%) had the prenatal diagnosis of an isolated congenital heart disease. The postmortem studies did not change the severity of these anomalies but were able to better characterize it in 11 cases.

In five cases of nonimmune hydrops fetalis no etiologic diagnosis was established, even after assessment of structural abnormalities of fetal heart, chromosome abnormalities, hemoglobinopathies, infections, and metabolic diseases (such as mucolipidosis I and II, galactosialidosis, mucopolysaccharidosis type I, IV, and VII, Gaucher disease type 2, gangliosidosis, Niemann-Pick disease type IA and C, multiple sulfatase deficiency, and sialic acid storage disease).

Infections were the cause of fetal abnormalities in five cases, cytomegalovirus in four cases, and parvovirus B19 in one case, as fetal hydrops.

Teratogens were identified in five cases. The use of misoprostol was the most common cause, found in 4 cases, and there was one warfarin embryopathy.

Other diagnoses were identified in 24 cases, such as acardiac fetus, umbilical cord abnormalities, placental abnormalities, cervical lymphangioma, severe decreased/absent fetal movement, and structural anomalies (bilateral micropthalmia, phocomelia of upper limbs, and spinal anomalies).

The anomalies are summarized in Table 1.

We have compared all the results from the obstetric, genetic, and fetal pathology evaluations for the presence or absence of agreement. Total agreement on the three areas was found in 165 cases (42%). The central nervous system anomalies were the main group with total agreement—70% (54/77 cases)—specifically the neural tube defects 81% (48/59 cases). Concordance in oligohydramnios sequence was 35% (6/17 cases). Prenatal diagnosis of familial syndromes had a total agreement in 84% (11/13 cases) of the cases. On the chromosome abnormality group we had a total of concordance of only 37%. All data were concordant in 38% of the prenatal diagnoses for advanced maternal age (29/77 cases).

Total concordance between obstetric data and genetic observation and/or laboratory analysis was present in 111 cases (29%). Total concordance between genetic findings and autopsy results was present in 116 cases (30%). In 100 cases (26%) there was total agreement between obstetric data and fetal pathology evaluation. 

Etiological diagnoses were changed or established after the genetic and/or autopsy observations in 80 cases (21%). After the genetic assessment (laboratorial results and fetal observation) the etiological diagnosis was changed in 49% (39/80 cases) of the cases. The postmortem results contributed to a modification of the diagnosis in 31% (25/80 cases) of the cases, and the identification of fetal infection in amniotic fluid changed the diagnosis in 6% (5/80 cases). The association between the autopsy findings and the data from the genetic evaluation allowed to identify the etiological diagnosis or to change it in 14% of the cases (11/80 cases).

There was only one case in which the prenatal prognosis was changed: in a twin pregnancy the diagnosis of one fetus with structural abnormalities and the other with hydrops being not confirmed in the latter.

## 4. Discussion

In the last years, several studies tried to correlate the prenatal diagnosis with the results provided by fetopathogical and genetics evaluation and to compare the impact of the new information to final diagnosis, prognosis, and genetic counseling. 

In our study, chromosome abnormalities were the most common diagnosis, representing 39% of cases. Advanced maternal age was the major reason of its realization (52%). Central nervous system anomalies (20%) and genetic syndromes (11%) were the most frequent diagnosis after chromosome abnormalities. Nonmonogenic nonchromosomal polimalformative syndromes without specific etiology were presented in 5.2% of cases. In this group the genetic and autopsy evaluations provided data that improved significantly the genetic counseling. Isolated congenital heart disease was found only in 4% and congenital infections in 1% of the cases.

Vaknin et al. found that chromosome abnormalities were the most common cause of TOP in 32% of the cases, followed by central nervous system in 27%, genetic syndromes in 5%, and (22 cases) fetal infection of the cases in 8% [1]. Amini et al. referred to 13.7% of chromosome abnormalities, 34.8% of central nervous system anomalies, and 3.7% of isolated congenital heart disease [13]. Boyd et al. found an abnormal karyotype in 46%, central nervous system abnormalities in 21%, monogenic disease in 8%, and isolated heart defect in 3% of the cases [7].

These studies and ours allow us to suggest a fairly constant prevalence of central nervous system anomalies and isolated congenital heart disease. There is a significant difference between the prevalence of genetic syndromes; in our opinion this value is dependent on the extent of the etiological investigation performed. There is a wide difference in the prevalence of chromosome abnormalities, which we relate to differences in the design of each study.

Genetic evaluation, laboratory tests, and fetus observation by a clinical geneticist are a reality in our department and proved to bring out valuable information. This was shown mainly in precise the diagnosis in many situations (49% of the cases in our study). 

Fetal postmortem studies were not always performed in other series. Boyd et al. showed that autopsy rates fell from 84% to 67% in 2000 [7]. Vaknin et al. had only 11% (52/462 cases) of cases submitted to fetopathological studies [1], and in Hungary study performed by Kaiser et al. 60% of cases had autopsy studies [12]. 

In our study, the importance of fetal pathology can be seen when we analyze its weight in the alteration of diagnosis by itself (31%) and in addition with the genetic evaluation (14%). So the collaboration of a clinical geneticist and a fetal pathologist in a prenatal diagnosis team in all tertiary centers is essential. Their participation improved the diagnostic skills in 45% of cases, in our department. This can be also supported by the study of Phadke and Gupta They found in their study that support from dysmorphologists and geneticists in fetal evaluation changed or improved the diagnosis in 33% of cases (13) and that is plays a crucial role in specific etiologic diagnosis and genetic counseling [11].

Full agreement between all data (ultrasound, genetics, and postmortem results) was obtained in 42% of cases in contrast with other studies. Phadke and Gupta referred to 72.5% of total concordance, Ramalho et al. 61.1%, Kaasen et al. 40%, Kaiser et al. 49%, and Boyd et al. 55% [4, 7, 8, 11, 12]. 

This discrepancy in the data can be attributed to the methodology used to comparison. In our study we considered all relevant data obtained by observation of the fetus by geneticist, in addition to the results of fetopathology, as able to change or improve the etiologic diagnosis. In these studies, except in that of Phadke et al., routine genetic evaluation isolated was not referred to as a source of information to diagnosis.

In only one situation the data changed the prognosis. Kaiser et al. referred to 2.4% of cases, where indication to TOP could not be supported after fetopathological results, Sankar and Phadke had 2 cases with total disagreement in 206 cases analyzed, and Phadke and Gupta also had 2 cases in 91examineted [2, 11, 12]. The data are summarized in Table 2.

The diagnostic accuracy was improved in 80 cases (21%) which corroborate the need of a multidisciplinary team in prenatal centers, as already noted by other authors [2, 3, 6, 11–13]. 

In conclusion, our study in concordance with others showed that when fetal anomalies have been detected by ultrasound, the specific diagnosis can be made or refined with association of genetic evaluation and autopsy studies. Altogether they affect favorably the genetic counseling and represent an important aspect to control the quality of information given to a couple previously to a TOP.

## Figures and Tables

**Table 1:** Anomalies found in 385 cases after TOP.

Anomalies	Number of cases
**Chromosome abnormalities**	**149**
**Numerical**	**122**
Trisomy 13	16
Trisomy 18	21
Trisomy 21	67
Monosomy X	12
Other	6
Structural unbalanced	22
Euploidies	5

**Nervous system anomalies**	**77**
Neural tube defects	59
Other	18

**Monogenic disorders**	**44**
**Familial syndromes**	**13**
Hemophilia A	3
X- fragile syndrome	2
Muscular spinal atrophy	1
Beta-thalassemia	1
Sickle cell anemia	1
Mucopolysaccharidosis type I	1
Gangliosidosis type I	1
Metachromatic leucodystrophy	1
Walker-Warburg syndrome	1
Familial amyloidotic polyneuropathy	1

**Diagnosis of index case**	**31**
Frontonasal dysplasia	3
Fryns syndrome	3
Meckel-Gruber syndrome	2
Multiple *pterygium* syndrome	2
Roberts syndrome	2
Bardet-Biedl syndrome	1
Thanatophoric dysplasia	1
Diastrophic dysplasia	1
Apert syndrome	1
Osteogenesis *imperfecta* type IIA	1
Tetraamelia with pulmonary agenesis	1
Bartsocas-Papas syndrome	1
Mucopolysaccharidosis type VII	1
Pfeiffer syndrome	1
Proteus syndrome	1
Atelosteogenesis type I	1
X-linked hydrocephalus	1
Solitary median maxillary central incisor syndrome	1
Other	6

**Sequences**	**37**
Oligohydramnios	17
Amnion rupture	6
Early urethral obstruction	6
Laterality sequences	2
Other	6

**Polymalformative syndrome**	**20**
**Isolated congenital heart disease**	**16**
**Nonimmune hydrops**	**5**
**Infections**	**5**
CMV	4
Parvovirus B19	1

**Teratogen**	**5**
Misoprostol	4
Warfarin	1

**Other diagnoses**	**24**

**Table 2:** Comparison of previous studies regarding total agreement and disagreement.

	**N**° of cases	Total agreement	Total disagreement
Phadke and Gupta [11]	91	72.5%	2,2% (2/91)
Ramalho et al. [8]	76	61.1%	0
Kaasen et al. [4]	228	58.4%	—
Kaiser et al. [12]	121	49%	2.4% (3/121)
Boyd et al. [7]	309	55%	—
Sankar and Phadke [2]	206	59%	1% (2/206)
Our study	385	42%	0.2% (1/385)
